# Association between anion gap and mortality in critically ill patients with gastrointestinal perforation: Evidence from the American MIMIC-IV and the Chinese Central-III Study

**DOI:** 10.1097/MD.0000000000050523

**Published:** 2026-09-04

**Authors:** Jixin Fu, Xiaoling Yin, De Zhang, Lei Luo, Junyang Kang

**Affiliations:** a Department of Gastrointestinal Surgery, Weihai Central Hospital Affiliated to Qingdao University, Weihai Central Hospital, Weihai, Shandong, China; b Department of Critical Care Medicine, Weihai Central Hospital Affiliated to Qingdao University, Weihai Central Hospital, Weihai, Shandong, China; c Department of Gastrointestinal Surgery, Danyang People’s Hospital, Zhenjiang, Jiangsu, China; d Department of Gastrointestinal Surgery, The Affiliated Hospital of Qingdao University, Weihai Central Hospital, Qingdao, Shandong, China.

**Keywords:** all-cause mortality, anion gap, critical care, gastrointestinal perforation, MIMIC-IV

## Abstract

Critically ill patients with gastrointestinal (GI) perforation present with complex, rapidly progressing conditions and high mortality. The impact of anion gap (AG) on mortality in these patients remains understudied, particularly across distinct geographic populations. This retrospective, multicenter cohort study analyzed 1167 critically ill patients with GI perforation from the American MIMIC-IV database (n = 552) and the Chinese Central-III cohort (n = 615). AG was measured using the first value obtained within 24 hours of intensive care unit admission. Patients were stratified by AG quartiles. Multivariable Cox regression assessed mortality risk, restricted cubic spline analysis evaluated nonlinearity, and receiver operating characteristic analysis identified optimal AG thresholds and quantified prognostic performance. Comprehensive subgroup analyses were performed. Mortality rates were comparable between cohorts (MIMIC-IV: 11.8% in-hospital, 24.6% 28-day; Central-III: 16.3% in-hospital, 24.8% 28-day). In both cohorts, the highest AG quartile showed significantly elevated mortality risk versus the lowest quartile (MIMIC-IV: aHR = 5.97 for in-hospital, aHR = 5.26 for 28-day; Central-III: aHR = 3.35 for in-hospital, aHR = 4.18 for 28-day). Restricted cubic spline revealed a consistent nonlinear AG-mortality relationship. Receiver operating characteristic analysis confirmed AG’s significant discriminatory power and identified optimal cutoff values in both populations. This first binational (US–China) multicenter analysis confirms that elevated AG levels independently predict increased mortality in critically ill patients with gastrointestinal perforation. The consistent findings across Western and Asian intensive care unit populations underscore AG’s robustness as a readily available prognostic marker in this high-risk patient group.

## 1. Introduction

Critically ill patients with gastrointestinal (GI) perforation are often admitted to the intensive care unit (ICU), posing a significant challenge for both surgeons and ICU physicians. GI perforation, a common surgical emergency, results from a full-thickness defect in the intestinal wall from multiple etiologies.^[1,2]^ The ensuing leakage of intestinal contents precipitates intra-abdominal infection, which can progress to sepsis, septic shock, and severe metabolic acidosis.^[3,4]^ Gastrointestinal perforation carries high mortality and typically necessitates emergency surgery. In the early 20th century, secondary peritonitis had a mortality rate as high as 90%.^[5,6]^ Despite modern advances in antibiotics, surgical techniques, imaging, and resuscitation, GI perforation in critically ill patients remains a major challenge, with mortality rates still ranging from 16.9% to 30%.^[7-10]^

The anion gap (AG), determined by the concentrations of measured anions and cations,^[11]^ is a comprehensive indicator of acid-base metabolism and can reflect the overall metabolic status of patients. As a routinely available and inexpensive parameter, AG is commonly measured in ICU settings for initial metabolic assessment. Elevated serum AG is frequently associated with severe acid-base imbalances and poor outcomes in critically ill patients with metabolic acidosis, particularly in conditions such as sepsis,^[12]^ cardiac arrest,^[13]^ and kidney dysfunction.^[14]^ We specifically focused on GI perforation patients because this population frequently develops severe intra-abdominal infections and sepsis, which often lead to significant metabolic acidosis – making AG a particularly relevant prognostic marker in this context. In adult ICU patients, AG has been shown to be a valuable tool with good sensitivity and specificity for predicting prognosis or mortality. Several studies have also linked high AG levels with poor outcomes in patients with acute pancreatitis,^[15]^ sepsis,^[16]^ acute pesticide poisoning,^[17]^ and other critical conditions. Additionally, AG has been used to predict mortality in pediatric ICU patients.^[18]^ However, despite the high prevalence of acid-base disorders and metabolic acidosis in patients with GI perforation, the specific prognostic utility of AG in this distinct clinical context remains unestablished. Furthermore, it is unknown whether this potential association remains consistent across diverse patient populations.

The aim of this study was to evaluate the association between AG levels and both in-hospital and 28-day mortality in critically ill patients with GI perforation, using data from the American MIMIC-IV cohort and the Chinese Central-III cohort.

## 2. Materials and methods

### 2.1. Study design and population

We utilized electronic health records from both a US public critical care database and a proprietary real-world dataset from China. The Medical Information Mart for Intensive Care (MIMIC-IV, version 2.2; Laboratory for Computational Physiology, Massachusetts Institute of Technology) provided data on 3,15,460 inpatients at Beth Israel Deaconess Medical Center from 2008 to 2019.^[19]^ Access to the database was certified for researcher Jixin Fu (Record ID 60228262), who performed data extraction. Inclusion criteria were: age ≥ 18 years, gastrointestinal perforation, and first ICU admission. Exclusion criteria included: absence of discharge status, ICU stay<24 hours, missing ICU anion gap data, and incomplete follow-up. A total of 522 patients with critical GI perforation were included from the MIMIC-IV database.

**Figure 1.**
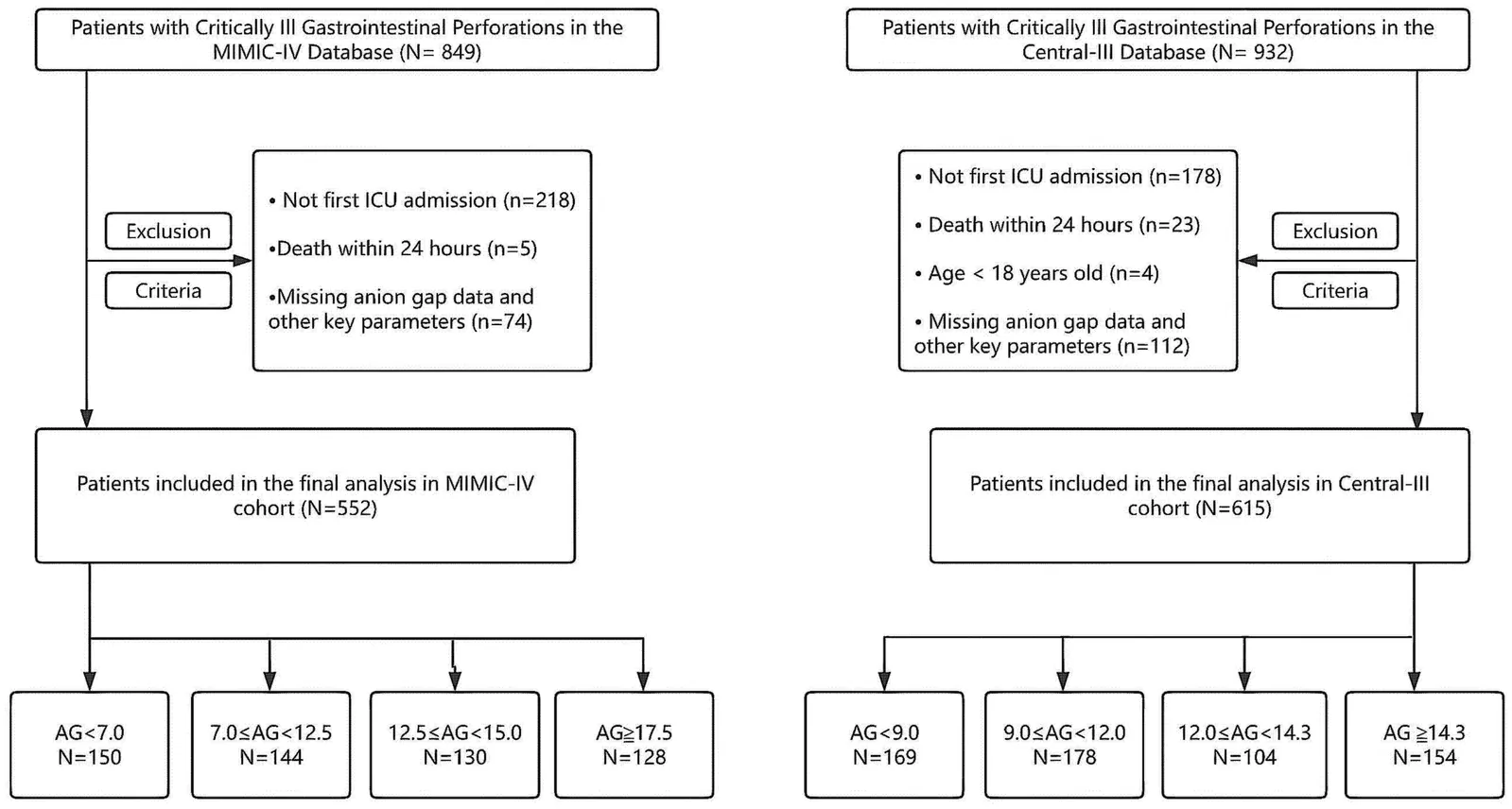
Flowchart of study design. ICU = intensive care unit, MIMIC-IV = Medical Information Mart for Intensive Care.

The Central-III cohort in China included patients from 3 tertiary teaching hospitals: Weihai Central Hospital, The Affiliated Hospital of Qingdao University, and Danyang People’s Hospital. Critically ill patients with gastrointestinal perforation from 2019 to 2024 were included, using the same inclusion and exclusion criteria as in the US cohort. A total of 615 participants were enrolled (Fig. 1).

The study was approved by the Ethics Committee of Weihai Central Hospital (Approval No. 2024-LL-273-01) and was conducted in accordance with the Declaration of Helsinki.

### 2.2. Data collection

Access to the MIMIC-IV database required completing a training course and passing the ‘Protecting Human Research Participants’ exam on the National Institutes of Health website (Certificate No. 12897170). We extracted variables from the MIMIC-IV database, including demographics, comorbidities, laboratory results (data were obtained within 24 hours of ICU admission), clinical indices, procedures, medications, and discharge status. The code for data query and extraction is publicly available on the repository (website: https://github.com/MIT-LCP/mimic-code).

In the Central-III cohort, in-hospital data were collected from the electronic clinical management system, which included demographic characteristics, clinical variables, and discharge status. Patient follow-up data post-discharge were collected from the follow-up databases of 3 hospitals, derived from telephone interviews or outpatient visits (see Table S1, Supplemental Digital Content 1 for details).

To address missing data, variables with more than 30% missing values were excluded from the analysis. For those with <30% missing, multiple imputation was applied to estimate missing values.^[20]^ This approach aimed to minimize bias and preserve the accuracy and reliability of the study findings.

Covariates for the multivariable model were preselected based on established clinical relevance to the outcome and data availability across the respective databases. The index measurement for all laboratory variables, including AG, was defined as the first value obtained within 24 hours of ICU admission. To preclude immortal time bias, the start of follow-up for time-to-event analyses (e.g., 28-day mortality) was aligned with the time of ICU admission, ensuring all baseline assessments preceded the outcome observation period. Non-laboratory covariates were extracted through structured retrospective review of medical records. For the MIMIC-IV cohort, these variables were sourced from pre-validated ICD codes and clinical notes, integral to the database’s standard curation protocol.

### 2.3. Outcomes and definitions

The primary outcomes of the study were in-hospital mortality and 28-day mortality. Comorbidities were identified using ICD-9 or ICD-10 codes from discharge diagnoses in the MIMIC-IV database, and ICD-10 codes in the Central-III cohort.

### 2.4. Statistical analysis

Patients were divided into 4 groups based on AG quartiles in both the MIMIC-IV and Central-III cohorts. Non-normally distributed variables were reported as weighted medians with interquartile ranges, while normally distributed variables were expressed as weighted means with standard errors. Categorical data were presented as counts and percentages. Group comparisons were performed using ANOVA, the Kruskal–Wallis test for continuous variables, or the chi-square test for categorical variables, as appropriate.

Univariable and multivariable Cox regression models were used to assess the association between AG levels and mortality in both cohorts. Variables with significant baseline differences or clinical relevance, common to both cohorts, were included in the final multivariable models. Model 1 was unadjusted; model 2 adjusted for age and gender; and model 3 further adjusted for age, gender, serum albumin, blood creatinine, sodium, potassium, vasoactive medication use, lactate, hemoglobin, white blood cell count, prothrombin time, simplified acute physiology score II (SAPS II), diabetes mellitus, and hypertension. Restricted cubic spline (RCS) analysis was applied to investigate the relationship between AG levels and all-cause mortality, adjusting for the same factors as the multivariable regression model. The results are reported as hazard ratios (HR) with 95% confidence intervals (CI). Subgroup analyses were also performed to evaluate the effect of AG on mortality within different subgroups, stratified by gender (male or female), age (≥60 or <60 years), diabetes (yes or no), and hypertension (yes or no).

To further evaluate the impact of AG levels on mortality in patients with critically ill GI perforation, we employed the bootstrap method to generate receiver operating characteristic (ROC) curves and calculated AUC values, comparing AG with the SAPS II, lactate levels, albumin, and red blood cell distribution width (RDW) – factors known to significantly affect mortality in this population. To this end, we further assessed the calibration of the model to gauge the robustness of the anion gap’s prognostic value.

Data analysis was performed using R software (version 4.2.2, R Project for Statistical Computing), with statistical significance set at *P* < .05 for 2-sided tests.

## 3. Results

### 3.1. Baseline characteristics

A total of 552 patients with critically ill GI perforation were included from the US MIMIC-IV cohort (mean age [SD]: 65.4 ± 16.4 years; 49% male) and 615 patients from the Chinese Central-III cohort (mean age [SD]: 66.5 ± 16.0 years; 75% male). Patients were stratified into 4 groups based on AG levels: in the MIMIC-IV cohort (quartile 1 [n = 150], AG < 7.0 mmol/L; quartile 2 [n = 144], 7.0 ≤ AG < 12.5 mmol/L; quartile 3 [n = 130], 12.5 ≤ AG < 15 mmol/L; quartile 4 [n = 128], AG ≥ 15 mmol/L) and in the Central-III cohort (quartile 1 [n = 169], AG < 9 mmol/L; quartile 2 [n = 178], 9 ≤ AG < 12 mmol/L; quartile 3 [n = 104], 12 ≤ AG < 14.3 mmol/L; quartile 4 [n = 154], AG ≥ 14.3 mmol/L). The designations Q1 through Q4 are used as group labels representing the progression from lowest to highest AG values, not as strict statistical quartiles.

There was a statistically significant difference in AG levels and AG classification between survivors and non-survivors of critically ill GI perforation in both the MIMIC-IV and Central-III cohorts (*P* < .001). Detailed baseline characteristics of both cohorts are provided in Table 1.

**Table 1:** Baseline demographic, clinical, and laboratory characteristics of the study cohorts.

	MIMIC-IV cohort							Central-III cohort						
Characteristics	Overall	In-hospital mortality		*P* value	28-d mortality		*P* value	Overall	In-hospital mortality		*P* value	28-d mortality		*P* value
		Yes	No		Yes	No			Yes	No		Yes	No	
	N = 552	N = 65	N = 487		N = 136	N = 416		N = 615	N = 100	N = 515		N = 153	N = 462	
Aniongap (mmol/L)	15.4 (4.0)	19.1 (5.4)	14.9 (3.5)	<.001	17.4 (4.7)	14.8 (3.5)	<.001	12.1 (4.7)	14.3 (5.3)	11.6 (4.4)	<.001	14.3 (5.2)	11.3 (4.2)	<.001
Aniongap classification				<.001			<.001				<.001			<.001
Quartile 1	150 (27%)	5 (7.7%)	145 (30%)		18 (13%)	132 (32%)		169 (27%)	13 (13%)	156 (30%)		20 (13%)	149 (32%)	
Quartile 2	144 (26%)	14 (22%)	130 (27%)		37 (27%)	107 (26%)		178 (29%)	18 (18%)	160 (31%)		28 (18%)	150 (32%)	
Quartile 3	130 (24%)	11 (17%)	119 (24%)		26 (19%)	104 (25%)		114 (19%)	27 (27%)	87 (17%)		39 (25%)	75 (16%)	
Quartile 4	128 (23%)	35 (54%)	93 (19%)		55 (40%)	73 (18%)		154 (25%)	42 (42%)	112 (22%)		66 (43%)	88 (19%)	
Basic characteristic														
Age (yr)	65 (16)	70 (14)	65 (16)	.005	71 (15)	64 (16)	<.001	67 (16)	67 (17)	66 (16)	.4	71 (16)	65 (16)	<.001
Gender				>.9			.3				.4			.028
Male	271 (49%)	32 (49%)	239 (49%)		61 (45%)	210 (50%)		463 (75%)	72 (72%)	391 (76%)		105 (69%)	358 (77%)	
Female	281 (51%)	33 (51%)	248 (51%)		75 (55%)	206 (50%)		152 (25%)	28 (28%)	124 (24%)		48 (31%)	104 (23%)	
Lab parameters														
Albumin (g/dL)	2.75 (0.69)	2.63 (0.74)	2.77 (0.68)	.2	2.66 (0.72)	2.78 (0.68)	.13	3.12 (0.72)	3.04 (0.81)	3.13 (0.70)	.3	2.97 (0.81)	3.17 (0.68)	.003
Bun (mg/dL)	28 (19)	39 (21)	26 (19)	<.001	36 (23)	25 (17)	<.001	26 (17)	31 (22)	25 (16)	.049	35 (23)	24 (14)	<.001
Creatinine (mg/dL)	1.43 (1.28)	1.84 (1.00)	1.38 (1.30)	<.001	1.63 (1.19)	1.37 (1.30)	.001	5.50 (4.31)	6.66 (4.87)	5.28 (4.16)	.003	7.27 (5.58)	4.92 (3.62)	<.001
Glucose (mg/dL)	142 (58)	146 (68)	142 (56)	>.9	140 (60)	143 (57)	.3	140 (64)	146 (68)	139 (63)	.5	144 (68)	139 (63)	.7
Sodium (mmol/L)	137.8 (4.5)	138.6 (4.9)	137.7 (4.5)	.054	138.1 (5.4)	137.8 (4.2)	.6	141.3 (51.9)	139.4 (4.4)	141.6 (56.7)	>.9	138.9 (4.7)	142.1 (59.8)	.14
Potassium (mmol/L)	4.24 (0.64)	4.31 (0.65)	4.24 (0.64)	.2	4.37 (0.64)	4.20 (0.63)	.002	4.41 (5.36)	4.29 (0.71)	4.44 (5.85)	.3	4.26 (0.78)	4.46 (6.17)	.5
ALT (U/L)	85 (289)	175 (486)	69 (235)	.004	139 (413)	62 (213)	.004	33 (65)	39 (71)	31 (63)	.047	37 (60)	31 (66)	.020
ALP (U/L)	105 (95)	98 (68)	106 (99)	.6	113 (94)	101 (95)	.12	61 (41)	61 (50)	61 (40)	.5	64 (53)	60 (38)	>.9
AST (U/L)	139 (474)	353 (905)	101 (337)	<.001	249 (742)	92 (288)	<.001	42 (120)	50 (128)	40 (118)	.052	46 (105)	40 (125)	.008
Total bilirubin (mg/dL)	1.53 (2.38)	1.95 (2.94)	1.45 (2.26)	.053	2.05 (3.10)	1.31 (1.96)	.043	1.13 (1.49)	1.17 (1.10)	1.13 (1.55)	.6	1.28 (1.22)	1.09 (1.57)	.3
INR	1.53 (0.58)	1.92 (0.90)	1.47 (0.50)	<.001	1.73 (0.77)	1.46 (0.49)	<.001	1.19 (0.48)	1.19 (0.23)	1.19 (0.52)	.2	1.23 (0.26)	1.18 (0.54)	<.001
PT (s)	16.7 (6.0)	20.5 (8.8)	16.2 (5.4)	<.001	18.6 (7.7)	16.0 (5.2)	<.001	15.54 (6.49)	13.16 (2.30)	16.01 (7.10)	.12	13.52 (2.59)	16.22 (7.50)	<.001
WBC count (10^9^/L)	11.6 (7.5)	12.3 (13.1)	11.5 (6.4)	.2	12.1 (10.6)	11.5 (6.2)	.5	11.4 (21.8)	11.5 (9.5)	11.3 (23.5)	.7	10.5 (8.4)	11.6 (24.7)	.3
PLT count (10^9^/L)	256 (122)	202 (100)	263 (122)	<.001	233 (120)	264 (121)	.004	242 (106)	241 (128)	242 (101)	.9	249 (132)	239 (96)	.8
Hemoglobin (mg/dL)	10.92 (1.87)	11.13 (2.06)	10.89 (1.84)	.4	10.82 (1.90)	10.96 (1.86)	.6	12.01 (2.50)	11.83 (2.78)	12.04 (2.45)	.4	11.70 (2.80)	12.11 (2.39)	.12
LYM percentage (%)	14 (11)	15 (13)	14 (10)	.4	13 (11)	14 (11)	.8	13 (31)	12 (12)	19 (73)	>.9	17 (59)	11 (12)	.7
MONO percentage (%)	5.85 (3.14)	6.26 (4.63)	5.80 (2.88)	.7	6.25 (3.95)	5.73 (2.81)	.2	4.63 (4.07)	4.69 (4.33)	4.28 (2.07)	.8	4.30 (2.55)	4.73 (4.43)	.3
NE percentage (%)	74 (15)	69 (16)	75 (14)	<.001	73 (15)	75 (15)	.10	85 (50)	85 (55)	83 (12)	.5	82 (13)	86 (57)	.4
RDW (%)	15.19 (1.87)	15.57 (1.59)	15.14 (1.90)	.014	15.83 (2.04)	14.99 (1.76)	<.001	22 (25)	23 (14)	22 (27)	.14	27 (46)	20 (13)	<.001
Lactic acid (mmol/L)	2.97 (2.26)	5.75 (3.75)	2.48 (1.39)	<.001	4.48 (3.39)	2.48 (1.44)	<.001	2.80 (2.37)	3.36 (2.91)	2.65 (2.19)	.2	7.34 (0.09)	7.39 (0.35)	.004
PH	7.33 (0.09)	7.25 (0.13)	7.35 (0.08)	<.001	7.31 (0.12)	7.34 (0.07)	.033	7.37 (0.31)	7.34 (0.09)	7.38 (0.33)	.2	3.55 (2.83)	2.43 (2.02)	<.001
Comorbidity														
Cardiovascular disease	112 (20%)	18 (28%)	94 (19%)	.11	39 (29%)	73 (18%)	.005	143 (23%)	26 (26%)	117 (23%)	.5	54 (35%)	89 (19%)	<.001
Cerebrovascular disease	34 (6.2%)	2 (3.1%)	32 (6.6%)	.4	10 (7.4%)	24 (5.8%)	.5	66 (11%)	12 (12%)	54 (10%)	.7	22 (14%)	44 (9.5%)	.093
Chronic lung disease	141 (26%)	21 (32%)	120 (25%)	.2	36 (26%)	105 (25%)	.8	183 (30%)	32 (32%)	151 (29%)	.6	58 (38%)	125 (27%)	.011
Diabetes	124 (22%)	16 (25%)	108 (22%)	.7	24 (18%)	100 (24%)	.12	49 (8.0%)	9 (9.0%)	40 (7.8%)	.7	13 (8.5%)	36 (7.8%)	.8
Renal disease	94 (17%)	13 (20%)	81 (17%)	.5	27 (20%)	67 (16%)	.3	47 (7.6%)	15 (15%)	32 (6.2%)	.002	22 (14%)	25 (5.4%)	<.001
Malignant cancer	107 (19%)	14 (22%)	93 (19%)	.6	37 (27%)	70 (17%)	.008	79 (13%)	11 (11%)	68 (13%)	.5	23 (15%)	56 (12%)	.4
Liver disease	83 (15%)	16 (24.2%)	67 (13.7%)	.064	29 (21.1%)	54 (13%)	.059	29 (4.7%)	6 (6.0%)	23 (4.5%)	.4	9 (5.9%)	20 (4.3%)	.4
Hypertension	225 (41%)	24 (37%)	201 (41%)	.5	52 (38%)	173 (42%)	.5	137 (22%)	22 (22%)	115 (22%)	>.9	34 (22%)	103 (22%)	>.9
ICU parameters														
SAPS II	43 (15)	56 (15)	41 (14)	<.001	51 (15)	40 (14)	<.001	43 (20)	52 (21)	39 (19)	<.001	51 (20)	38 (19)	<.001
Vasoactive drugs	245 (44%)	56 (86%)	189 (39%)	<.001	88 (65%)	157 (38%)	<.001	169 (28%)	45 (45%)	124 (24%)	<.001	70 (46%)	99 (21%)	<.001

ALP = alkaline phosphatase, ALT = alanine aminotransferase, AST = aspartate transaminase, INR = international normalized ratio, LYM = lymphocyte, MONO = monocytes, NE = neutrophil, PLT count = platelet count, PT = prothrombin time, RDW = red blood cell distribution width, SAPS II = simplified acute physiology score II, WBC count = white blood cell count.

### 3.2. AG level and mortality

In the American MIMIC-IV cohort, there were 65 (11.8%) in-hospital mortalities and 136 (24.6%) 28-day mortalities, with the highest rates in quartile 4 (35 in-hospital mortalities [53.8%] and 55 28-day mortalities [40.4%]). The Chinese Central-III cohort showed similar trends, with 100 (16.3%) in-hospital mortalities and 153 (24.8%) 28-day mortalities, also highest in quartile 4 (42 in-hospital mortalities [42.0%] and 66 28-day mortalities [43.1%]).

After adjusting for confounders in the MIMIC-IV cohort, patients with an AG ≥ 15 mmol/L (quartile 4) had a significantly higher risk of both in-hospital (HR = 5.97, 95% CI: 1.59–27.9, *P* = .038) and 28-day (HR = 5.26, 95% CI: 1.75–17.3, *P* = .020) all-cause mortality compared to those with an AG < 7.0 mmol/L (quartile 1; Table 2). Similar findings were observed in the Chinese Central-III cohort, where patients with an AG ≥ 14.3 mmol/L (quartile 4) had an increased risk of in-hospital (HR = 3.35, 95% CI: 1.62–7.29, *P* < .001) and 28-day (HR = 4.18, 95% CI: 2.21–8.13, *P* < .001) all-cause mortality compared to quartile 1 (Table 2). Multicollinearity diagnostics using variance inflation factors confirmed no concerning collinearity among covariates in both cohorts, with all variance inflation factors values below 3.0.

**Table 2:** HRs (95% CI) for in-hospital mortality and 28-day mortality according to anion gap quartiles in patients with critically ill gastrointestinal perforation in the MIMIC-IV cohort and the Central-III cohort.

	Aniongap classification				*P*-trend
	Quartile 1	Quartile 2	Quartile 3	Quartile 4	
MIMIC-IV cohort					
In-hospital mortality					
Number of deaths/total	5/150	14/144	11/130	35/128	
Model 1	1.00 [Reference]	3.12 (1.16, 9.93)	2.68 (0.94, 8.75)	10.9 (4.48, 32.8)	<.001
Model 2	1.00 [Reference]	2.99 (1.12, 9.30)	2.53 (0.91, 8.07)	10.7 (4.42, 31.4)	<.001
Model 3	1.00 [Reference]	2.86 (0.79, 12.9)	1.90 (0.43, 9.66)	5.97 (1.59, 27.9)	.038
28-d mortality					
Number of deaths/total	8/150	37/144	26/130	55/128	
Model 1	1.00 [Reference]	2.54 (1.38, 4.81)	1.83 (0.96, 3.58)	5.53 (3.07, 10.4)	<.001
Model 2	1.00 [Reference]	2.50 (1.35, 4.78)	1.73 (0.89, 3.41)	5.63 (3.08, 10.7)	<.001
Model 3	1.00 [Reference]	3.83 (1.38, 11.8)	2.77 (0.93, 8.90)	5.26 (1.75, 17.3)	.020
Central-III cohort					
In-hospital mortality					
Number of deaths/total	13/169	18/178	27/104	42/154	
Model 1	1.00 [Reference]	1.35 (0.64, 2.91)	3.72 (1.86, 7.82)	4.50 (2.36, 9.11)	<.001
Model 2	1.00 [Reference]	1.37 (0.65, 2.96)	3.77 (1.87, 7.96)	4.49 (2.34, 9.13)	<.001
Model 3	1.00 [Reference]	1.36 (0.62, 3.03)	3.82 (1.84, 8.34)	3.35 (1.62, 7.29)	<.001
28-d mortality					
Number of deaths/total	20/169	28/178	39/104	66/154	
Model 1	1.00 [Reference]	1.39 (0.75, 2.61)	3.87 (2.13, 7.23)	5.59 (3.22, 10.1)	<.001
Model 2	1.00 [Reference]	1.45 (0.78, 2.77)	4.28 (2.32, 8.13)	5.29 (3.01, 9.65)	<.001
Model 3	1.00 [Reference]	1.47 (0.77, 2.86)	4.44 (2.33, 8.72)	4.18 (2.21, 8.13)	<.001

Data are presented as HR (95% CI) unless indicated otherwise; model 1 was unadjusted; model 2 was adjusted for age (continuous) and sex (male or female); and model 3 was adjusted for model 1 plus serum albumin, blood creatinine, blood sodium, blood potassium, vasoactive medication use, lactate, hemoglobin, white blood cell counts, prothrombin time, SAPS II, diabetes mellitus, and hypertension.

CI = confidence interval, HR = hazard ratio, MIMIC-IV = Medical Information Mart for Intensive Care, SAPS II = SAPS II = simplified acute physiology score II.

### 3.3. Restricted cubic spline analysis

**Figure 2.**
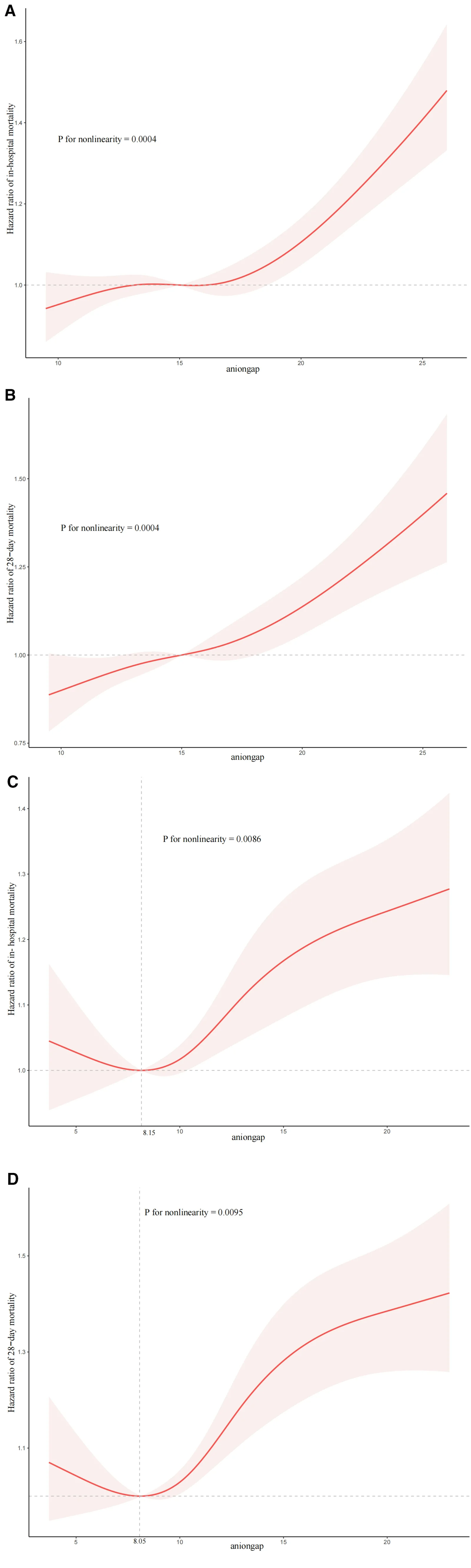
Restricted cubic spline analysis of the association between anion gap (AG, mmol/L) and mortality risks in critically ill patients with gastrointestinal perforation. Analyses were adjusted for (list key adjusted variables, e.g., age, sex, comorbidities). (A) In-hospital mortality, MIMIC-IV cohort; (B) 28-day mortality, MIMIC-IV cohort; (C) In-hospital mortality, Central-III cohort; (D) 28-day mortality, Central-III cohort. MIMIC-IV = Medical Information Mart for Intensive Care.

RCS analysis revealed a nonlinear association between AG levels and all-cause mortality in the American MIMIC-IV cohort (nonlinear *P* = .0004 for both in-hospital and 28-day mortality). Similar results were observed in the Chinese Central-III cohort, where AG levels were nonlinearly associated with in-hospital and 28-day mortality, with nonlinear *P*-values of .0086 and .0095, respectively (Fig. 2A, B). Additionally, we identified that the critical AG levels associated with the lowest risk of in-hospital and 28-day mortality in the Central-III cohort were 8.15 and 8.05, respectively (Fig. 2C, D).

### 3.4. Subgroup analyses

Subgroup analyses based on age, sex, hypertension, and diabetes status were conducted in both cohorts to evaluate the association between AG levels and all-cause mortality in patients with critically ill GI perforation. It should be emphasized that these analyses were exploratory in nature; therefore, the consistent pattern of association is the key takeaway, while individual *P*-values should be interpreted with caution. Among nondiabetic patients, AG levels were significantly associated with 28-day mortality in both the US MIMIC-IV cohort (*P* for trend = .023) and the Chinese Central-III cohort (*P* for trend = .011), as well as with in-hospital mortality in the Chinese Central-III cohort (*P* for trend = .047). This association was not observed in diabetic patients. No significant differences in concordance between the 2 cohorts were found in other subgroup analyses (Table 3). Additionally, in the Chinese Central-III cohort, the subgroup analysis of 28-day mortality by hypertension status showed an interaction *P*-value of .018, while the interaction *P*-values for other subgroup analyses were >.05 (Table 3).

**Table 3:** Subgroup analysis of anion gap quartiles, in-hospital mortality, and 28-day mortality in patients with critically ill gastrointestinal perforation in the MIMIC-IV cohort and the Central-III cohort.

	MIMIC-IV cohort						Central-III cohort					
	Aniongap classification				*P* for trend	*P* for interaction	Aniongap classification				*P* for trend	*P* for interaction
	Quartile 1	Quartile 2	Quartile 3	Quartile 4			Quartile 1	Quartile 2	Quartile 3	Quartile 4		
In-hospital mortality												
Age						.908						.850
<60 yr	1.00 [Reference]	3.59 (0.31, 69.4)	1.13 (0.05, 26.8)	10.2 (0.78, 23.7)	.200		1.00 [Reference]	2.45 (0.11, 44.4)	3.33 (0.15, 36.3)	11.2 (0.48, 55.7)	.380	
≥60 yr	1.00 [Reference]	3.91 (0.68, 35.8)	2.41 (0.32, 25.1)	5.03 (0.80, 47.4)	.300		1.00 [Reference]	0.91 (0.01, 59.3)	0.82 (0.01, 54.8)	13.1 (1.21, 602)	**.016**	
Sex						.405						.316
Male	1.00 [Reference]	3.69 (0.89, 21.4)	2.39 (0.46, 15.6)	9.93 (2.33, 59.0)	.011		1.00 [Reference]	0.82 (0.02, 26.0)	1.24 (0.54, 13.2)	5.05 (0.31, 159)	.110	
Female	1.00 [Reference]	1.75 (1.44, 7.17)	1.79 (0.63, 4.52)	3.59 (2.39, 16.4)	.049		1.00 [Reference]	1.21 (0.94, 5.71)	1.34 (0.88, 3.42)	5.15 (1.44, 10.41)	.059	
Diabetes						.707						.221
Yes	1.00 [Reference]	2.39 (0.79, 27.1)	2.07 (0.56, 13.2)	4.43 (1.23, 19.2)	.083		1.00 [Reference]	3.10 (0.66, 20.7)	2.66 (0.96, 7.27)	3.33 (1.67, 15.5)	.055	
No	1.00 [Reference]	2.16 (0.53, 10.4)	1.35 (0.25, 7.60)	2.90 (0.65, 15.0)	.500		1.00 [Reference]	0.72 (0.11, 3.92)	3.36 (0.72, 15.7)	3.70 (1.01, 15.8)	**.047**	
Hypertension						.658						.101
Yes	1.00 [Reference]	5.65 (0.62, 107)	0.95 (0.04, 23.4)	6.78 (0.74, 126)	.200		1.00 [Reference]	2.25 (0.12, 35.8)	056 (0.22, 15.4)	5.67 (0.88, 45.1)	.340	
No	1.00 [Reference]	1.65 (0.27, 11.9)	2.17 (0.34, 16.1)	5.85 (1.11, 40.1)	.140		1.00 [Reference]	0.39 (0.01, 3.75)	4.54 (0.88, 27.1)	7.60 (1.81, 40.3)	**.002**	
28-d mortality												
Age						0.787						.737
<60 yr	1.00 [Reference]	2.59 (0.36,21.9)	0.93 (0.13,7.28)	1.35 (0.15, 12.4)	0.700		1.00 [Reference]	1.29 (0.22,31.2)	0.62 (0.33,12.8)	4.33 (0.25, 23.4)	.650	
≥60 years	1.00 [Reference]	6.65 (1.78, 31.4)	3.90 (0.91, 19.8)	9.03 (2.17, 45.8)	**0.012**		1.00 [Reference]	1.03 (0.03, 72.9)	3.77 (0.18, 238)	13.1 (1.15, 697)	.053	
Sex						0.592						.290
Male	1.00 [Reference]	4.39 (1.27, 18.8)	2.20 (0.53, 10.30	7.40 (1.88, 34.9)	**0.021**		1.00 [Reference]	0.70 (0.01, 39.7)	3.58 (0.12, 172)	2.86 (0.18, 106)	.700	
Female	1.00 [Reference]	3.17 (0.76, 14.8)	2.94 (0.71, 13.9)	2.87 (0.55, 16.4)	0.400		1.00 [Reference]	2.76 (0.88, 11.1)	3.04 (0.88, 12.1)	4.07 (0.95, 17.0)	.458	
Diabetes						0.252						.269
Yes	1.00 [Reference]	0.49 (0.01, 45.9)	0.96 (0.03, 67.9)	3.90 (0.15, 332)	0.500		1.00 [Reference]	0.71 (0.11, 33.1)	0.59 (0.06, 57.5)	3.22 (0.27, 66.2)	.578	
No	1.00 [Reference]	5.58 (1.74, 20.7)	3.33 (0.96, 12.9)	5.32 (1.46, 21.6)	**0.023**		1.00 [Reference]	0.92 (0.24, 3.37)	5.47 (1.50, 21.4)	3.46 (1.11, 11.6)	**.011**	
Hypertension						0.531						**.018**
Yes	1.00 [Reference]	6.85 (1.26, 50.1)	1.82 (0.24, 14.0)	13.9 (2.32, 110)	**0.013**		1.00 [Reference]	0.63 (0.06, 6.25)	0.08 (0.01, 2.69)	0.03 (0.01, 1.20)	.200	
No	1.00 [Reference]	2.73 (0.65, 13.3)	3.33 (0.81, 16.0)	3.58 (0.84, 17.7)	0.3		1.00 [Reference]	0.92 (0.15, 5.08)	11.7 (2.50, 64.7)	10.7 (2.72, 50.7)	**<.001**	

Data are presented as HR (95% CI) unless indicated otherwise; the model was adjusted for age (continuous), sex (male or female), serum albumin, blood creatinine, blood sodium, blood potassium, vasoactive medication use (yes or no), lactate, hemoglobin, white blood cell counts, prothrombin time, SAPS II, diabetes mellitus (yes or no), and hypertension (yes or no). *P*-values < .05 are labeled in bold font.

CI = confidence interval, HR = hazard ratio, MIMIC-IV = Medical Information Mart for Intensive Care, SAPS II = SAPS II = simplified acute physiology score II.

### 3.5. ROC curve analysis

**Figure 3.**
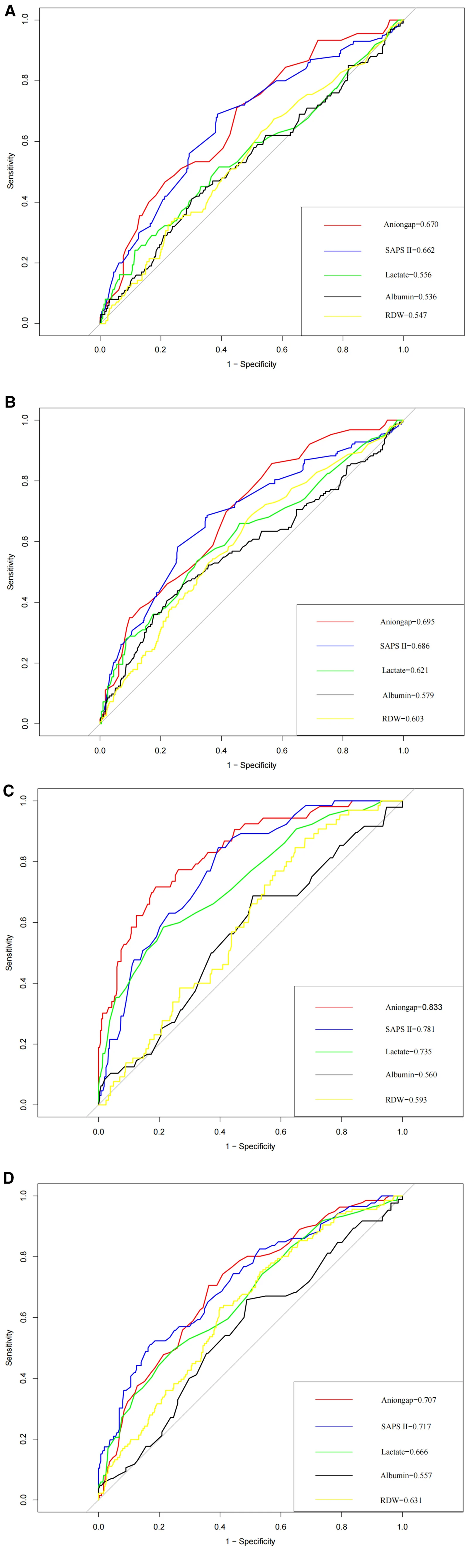
Receiver operating characteristic (ROC) curves evaluating the predictive performance of anion gap (AG, mmol/L) for all-cause mortality. Models were adjusted for age, sex, hypertension, and diabetes status. (A) In-hospital mortality, MIMIC-IV cohort; (B) 28-day mortality, MIMIC-IV cohort; (C) In-hospital mortality, Central-III cohort; (D) 28-day mortality, Central-III cohort. RDW = red blood cell distribution width, ROC = receiver operating characteristic, SAPS II = simplified acute physiology score II.

ROC analysis in the Chinese Central-III cohort showed that the predictive performance of AG for both in-hospital and 28-day mortality in critically ill GI perforation patients was comparable to that of the SAPS II score, with AUC values of 0.833 (95% CI: 0.729–0.906) and 0.707 (95% CI: 0.615–0.774), respectively, outperforming lactate, albumin, and RDW (Fig. 3A, B). Similarly, in the MIMIC-IV cohort, the AUC values for AG were 0.670 (95% CI: 0.579–0.761) for in-hospital mortality and 0.695 (95% CI: 0.615–0.774) for 28-day mortality, also exceeding those of other biomarkers (Fig. 3C, D). Furthermore, the calibration curve demonstrated the robust prognostic value of AG in critically ill patients with gastrointestinal perforation (Fig. S1, Supplemental Digital Content 2).

## 4. Discussion

To the best of our knowledge, this is the first study to investigate the relationship between AG levels and in-hospital as well as 28-day all-cause mortality in patients with critically ill GI perforations. To achieve this, we compared the consistency of findings between the American MIMIC-IV cohort and the Chinese Central-III cohort. Our results indicate that: elevated AG levels were associated with higher in-hospital and 28-day all-cause mortality in critically ill GI perforation patients in both cohorts; in the Chinese Central-III cohort, the lowest in-hospital and 28-day mortality rates occurred at AG levels of 8.15 and 8.05 mmol/L, respectively; and ROC analysis confirmed that AG levels show significant discriminatory power for all-cause mortality in these patients. Most importantly, this study identifies AG as a simple and effective biomarker for assessing mortality risk in critically ill GI perforation patients admitted to the ICU.

The AG, introduced by James Gamble in 1936, only became widely applicable with the advent of automated electrolyte analyzers in the mid-20th century.^[21]^ It remains a valuable tool for detecting complex acid-base disorders and facilitating prompt differential diagnosis, particularly in critically ill patients. Accumulating evidence supports its prognostic utility: elevated AG has been independently associated with increased mortality in sepsis,^[22]^ acute myocardial infarction,^[23]^ and following coronary artery bypass grafting.^[24]^ Similar associations have been observed in chronic kidney disease,^[25]^ aortic aneurysm,^[26]^ and COVID-19, where AG emerged as a key predictor of mortality in a machine learning model.^[27]^

The impact of AG levels on critically ill patients with GI perforation remains unclear. Given that GI perforations in critically ill patients, often leading to sepsis, are marked by hemodynamic instability, severe hypoxemia, and metabolic acidosis, we hypothesized a significant association between AG levels and patient prognosis. To account for potential racial differences, we used both the American MIMIC-IV and Chinese Central-III databases for validation. Our findings demonstrated that elevated AG levels were significantly associated with in-hospital and 28-day mortality in critically ill GI perforation patients, independent of racial differences.

Several parameters are currently effective in predicting the prognosis of critically ill patients with GI perforation. Chiarugi et al demonstrated that APACHE II and SAPS scores were strongly associated with mortality in patients with perforated peptic ulcers.^[28]^ Similarly, Kang et al identified postoperative lactate levels as the most powerful predictor of in-hospital mortality in GI perforation patients.^[29]^ Other studies have also linked prognosis to albumin levels^[30]^ and RDW.^[31]^

To evaluate the predictive value of AG levels for short-term all-cause mortality in critically ill GI perforation, we calculated the area under the curve (AUC) from the ROC analysis. AG levels showed predictive power comparable to SAPS II and outperformed other parameters in both the American MIMIC-IV and Chinese Central-III cohorts. These findings suggest that AG is a valuable prognostic marker for critically ill GI perforation patients in the ICU. Additionally, in the Central-III cohort, the lowest in-hospital and 28-day mortality rates were observed at AG levels of 8.15 and 8.05 mmol/L, respectively. However, these findings require further validation to account for potential racial differences and reduce bias.

The pathophysiologic mechanisms linking AG levels and mortality in critically ill GI perforation patients remain poorly understood. The condition triggers a “triple-hit” model comprising chemical peritonitis from digestive enzyme leakage, polymicrobial sepsis, and massive third-spacing with capillary leak. These insults collectively stimulate the release of inflammatory cytokines such as interleukin-1 and interleukin-6, triggered by the translocation of gram-negative bacteria, leading to increased anaerobic fermentation. This process produces elevated levels of phosphates, sulfates, and organic acids, including lactic acid, contributing directly to the rise in AG levels.^[32]^ An acidic extracellular microenvironment can trigger an inflammatory response, including neutrophil activation and complement system engagement, leading to systemic inflammatory response syndrome and rapid progression to sepsis and shock^33-35]^ This suggests that elevated AG levels may serve as an early marker of critical illness, offering a “window of time” for intervention before sepsis develops in patients with GI perforation. Additionally, previous studies indicate that elevated AG is primarily driven by metabolic acidosis, with elevated serum lactate being a common contributor.^[28,36,37]^ However, several studies have disputed the correlation between serum lactate and AG.^[12,36-38]^ To investigate this further, we performed a linear correlation analysis, which revealed correlation coefficients of 0.603 and 0.371 between serum lactate and AG in the MIMIC-IV and Central-III cohorts, respectively, indicating no significant linear relationship (Fig. S2, Supplemental Digital Content 3). Additionally, our study showed that elevated AG remained associated with increased mortality even after adjusting for serum lactate levels. These findings suggest that the impact of AG on mortality in critically ill GI perforation is independent of serum lactate.

The J-shaped relationship between AG and mortality in our study, identified through our RCS analysis, underscores the dual metabolic perils faced by critically ill patients with gastrointestinal perforation. The risk nadir at AG levels of 8.05 to 8.15 mmol/L demarcates a zone of relative metabolic homeostasis. Beyond this range, the AG reflects opposing yet deleterious states. Elevated AG is a direct marker of metabolic acidosis driven by unmeasured anions, principally lactate from profound tissue hypoperfusion – a hallmark of septic shock and a key mortality driver. On the opposite end, a low AG often implicates severe hypoalbuminemia, which not only reflects chronic disease burden and inflammatory status but can also compromise oncotic pressure and drug binding, exacerbating critical illness. Thus, AG integrates multiple metabolic signals, and its extremes, in either direction, are biomarkers of distinct pathophysiological processes that converge on an elevated risk of death.

This study identified a significant association between AG levels and all-cause mortality in critically ill patients with GI perforation, a population characterized by unique pathophysiology including rapid peritoneal contamination and a high risk of polymicrobial sepsis. To our knowledge, this is the first large multicenter study to specifically establish AG as a prognostic marker in this distinct clinical context. The robustness of this association is further strengthened by its consistent replication across 2 independent binational cohorts – the US MIMIC-IV and Chinese Central-III databases – which differ in ethnicity, healthcare systems, and clinical practices. This consistency not only validates the relationship internally but also suggests that the AG–mortality link is a generalizable biological phenomenon, transcending regional medical variations.

Given that critically ill GI perforation patients frequently experience hemodynamic instability and severe acid-base imbalances, our findings carry direct clinical relevance. The anion gap, as a rapid and routinely available parameter, holds potential as a practical complement to established scoring systems such as SAPS-II. While SAPS-II offers a comprehensive baseline risk assessment at ICU admission, serial AG measurements could provide dynamic, real-time insight into evolving metabolic acidosis and the burden of unmeasured anions – enabling earlier detection of clinical deterioration. Integrating AG with SAPS-II may thus improve risk stratification, helping clinicians identify high-risk patients who might benefit from more aggressive resuscitation or intensified monitoring.

We acknowledge several limitations in this study. First, as a consequence of the retrospective design, our findings should be interpreted as identifying associations rather than establishing causality. Despite this, our study is the first real-world analysis to demonstrate a significant association between elevated AG levels and short-term all-cause mortality in critically ill GI perforation patients, with consistent findings across both American and Chinese cohorts. Second, while the sample size was relatively small, the inclusion of patients from multiple centers and ethnicities enhances the generalizability of our results. Third, the Chinese Central-III cohort lacked SOFA score data, a key sepsis risk assessment tool. As a substitute, we used the SAPS-II score, which reliably identifies mortality risk in ICU patients.^[39]^ Nevertheless, it is important to acknowledge that the absence of SOFA scores, along with other unmeasured confounders such as sepsis severity, surgical delays, renal function assessment, drug effects, missing microbiology data, and fluid management, may represent a residual source of bias in our findings. Fourth, the inclusion of 2 distinct cohorts introduces inherent heterogeneity in patient characteristics and healthcare systems across countries and ethnic groups, which may act as unmeasured confounding factors. Consequently, our findings warrant further validation through large-scale, prospective studies. Finally, our study focused only on short-term outcomes (in-hospital and 28-day mortality); further research is needed to examine the long-term relationship between AG levels and critically ill GI perforation outcomes.

## 5. Conclusion

This study establishes a consistent nonlinear relationship between AG levels and both in-hospital and 28-day mortality in critically ill patients with gastrointestinal perforation across American and Chinese cohorts. AG represents a readily available early warning indicator for risk stratification in the ICU setting. Future research should validate these findings through large-scale randomized trials assessing AG-guided management and investigate the prognostic significance of dynamic AG monitoring.

## Acknowledgments

We extend our gratitude to those who contributed to the MIMIC-IV database for this study. We also thank the doctors and nurses of the Department of Intensive Care Medicine at Weihai Central Hospital, The Affiliated Hospital of Qingdao University, and Danyang People’s Hospital for their exceptional care of the patients and unwavering support for this research.

## Author contributions

**Conceptualization:** Jixin Fu, Junyang Kang.

**Data curation:** Jixin Fu, Xiaoling Yin, De Zhang, Lei Luo.

**Formal analysis:** Jixin Fu, Xiaoling Yin.

**Methodology:** Xiaoling Yin, De Zhang, Lei Luo, Junyang Kang.

**Project administration:** Junyang Kang.

**Supervision:** Junyang Kang.

**Writing – original draft:** Jixin Fu.

**Writing – review & editing:** Junyang Kang.










