# Supplementary material for: Association between anion gap and mortality in critically ill patients with gastrointestinal perforation: Evidence from the American MIMIC-IV and the Chinese Central-III Study
Source: Medicine (Baltimore). 2026 Sep 4;105(36):e50523. doi: 10.1097/MD.0000000000050523 (PMC13549532; doi:10.1097/MD.0000000000050523)
Supplement: Supplementary file 1 [file medi-105-e50523-s001.docx]

Table S1 Follow-up register of discharged patients

| Name | Gender | Age | phone number | ID | Discharge time | Discharge diagnosis | Follow-up time | | | Current health status | | | | lifestyle | | | medication | Suggestions for us | follow-up time | follow-up physician | comment |
| --- | --- | --- | --- | --- | --- | --- | --- | --- | --- | --- | --- | --- | --- | --- | --- | --- | --- | --- | --- | --- | --- |
|  |  |  |  |  |  |  | 2 weeks | 1 month | 3 months | Asymptomatic | New symptoms appear | Original symptoms persist | Death | Smoking | Drinking | moderate exercise |  |  |  |  |  |
|  |  |  |  |  |  |  |  |  |  |  |  |  |  |  |  |  |  |  |  |  |  |
|  |  |  |  |  |  |  |  |  |  |  |  |  |  |  |  |  |  |  |  |  |  |
|  |  |  |  |  |  |  |  |  |  |  |  |  |  |  |  |  |  |  |  |  |  |
|  |  |  |  |  |  |  |  |  |  |  |  |  |  |  |  |  |  |  |  |  |  |
|  |  |  |  |  |  |  |  |  |  |  |  |  |  |  |  |  |  |  |  |  |  |
|  |  |  |  |  |  |  |  |  |  |  |  |  |  |  |  |  |  |  |  |  |  |
|  |  |  |  |  |  |  |  |  |  |  |  |  |  |  |  |  |  |  |  |  |  |
|  |  |  |  |  |  |  |  |  |  |  |  |  |  |  |  |  |  |  |  |  |  |
|  |  |  |  |  |  |  |  |  |  |  |  |  |  |  |  |  |  |  |  |  |  |
|  |  |  |  |  |  |  |  |  |  |  |  |  |  |  |  |  |  |  |  |  |  |
|  |  |  |  |  |  |  |  |  |  |  |  |  |  |  |  |  |  |  |  |  |  |
|  |  |  |  |  |  |  |  |  |  |  |  |  |  |  |  |  |  |  |  |  |  |
|  |  |  |  |  |  |  |  |  |  |  |  |  |  |  |  |  |  |  |  |  |  |
|  |  |  |  |  |  |  |  |  |  |  |  |  |  |  |  |  |  |  |  |  |  |

Exercise time is approximately equal to 15 minutes or more per day √, otherwise x.

Patients' discharge survival status was determined by reviewing hospital discharge records. The 28-day survival status was assessed using follow-up data from tertiary care hospitals. Each hospital maintained a database of discharged patients' follow-up visits, which were accessed to confirm 28-day survival status. **Gastrointestinal Surgery Department of Weihai Central Hospital Follow-up Protocol:** Patients discharged from the Gastrointestinal Surgery Department of Weihai Central Hospital were followed up either through outpatient clinic visits during the second week post-discharge or via telephone for those unable to attend. **Gastrointestinal Surgery Department, Affiliated Hospital of Qingdao University, Qingdao, China Follow-up Protocol:** Patients discharged from the Gastrointestinal Surgery Department, Affiliated Hospital of Qingdao University, were followed up via telephone at the 2nd week, 1st month, 3rd month, and 6th month post-discharge. **General Surgery Department, Danyang City People's Hospital Follow-up Protocol:** Patients discharged from the General Surgery Department at Danyang City People's Hospital were followed up via telephone at the 2nd week, 1st month, 3rd month, and 6th month post-discharge. We determined patient survival status on day 28 by reviewing the follow-up database of those three hospitals. Follow-up analysis was also performed.
